# Development and validation of a CT-based predictive model for new vertebral compression fractures: the role of vertebral CT attenuation and paraspinal muscle

**DOI:** 10.3389/fmed.2026.1860827

**Published:** 2026-07-22

**Authors:** Yiqi Wu, Qing Song, Canwei Hu, Chaoyi Yin, Zhiqing Chen, Pujian Zhong, Binshan Zhang

**Affiliations:** Dongguan Hospital of Guangzhou University of Chinese Medicine, Dongguan, China

**Keywords:** computed tomography, LASSO, new vertebral compression fracture, nomogram, paraspinal muscle, percutaneous vertebroplasty, vertebral CT attenuation

## Abstract

**Objectives:**

Osteoporotic vertebral compression fractures (OVCFs) are a major health burden, especially with aging populations. While percutaneous vertebroplasty (PVP) is effective, new vertebral compression fractures (NVCFs) are a common complication. Current NVCF prediction models focus on cement factors or bone density alone, ignoring the critical role of paraspinal muscles. To address this gap, our study integrates preoperative CT imaging of bone density and muscle area with clinical data to develop a visualized early prediction model for NVCF risk, aiming to improve patient outcomes.

**Methods:**

A total of 423 patients who underwent PVP at Guangzhou University of Chinese Medicine Dongguan hospital were retrospectively analyzed and were allocated into a training set and a validation set in an 8:2 ratio. The study employed multivariate logistic regression analysis and the Least Absolute Shrinkage and Selection Operator (Lasso) to develop predictive models and generate nomogram. Receiver Operating Characteristic (ROC) curves and calibration curves were plotted to evaluate the models’ discriminatory and calibration capabilities, Decision Curve Analysis (DCA) and Clinical Impact Curve(CIC)were used to assess the models’ clinical applicability and utility.

**Results:**

Lasso regression analysis identified age, albumin (ALB), paravertebral muscle area, bone CT value, low-energy trauma, and single-segment fracture as significant predictors. AUC of the nomogram model in the training set was 0.893 (95% CI: 0.858–0.928) and in the validation set was 0.952 (95% CI: 0.903–1), demonstrating its robust predictive capability. DCA and CIC further suggest that this nomogram model possesses substantial clinical application value.

**Conclusion:**

The nomogram developed based on conventional clinical data in this study, has undergone internal validation and demonstrated efficacy in predicting NVCF following PVP. It serves as a valuable decision-making tool for clinicians. However, further multicenter studies are required for external validation to confirm its generalizability.

## 1 Introduction

Osteoporotic vertebral compression fractures (OVCFs) are one of the leading causes of death and disability among older adults worldwide (1). With an aging population, the prevalence of osteoporosis and fractures in China is expected to continue rising (2). Percutaneous vertebroplasty (PVP) stabilizes vertebral compression fractures and rapidly alleviates symptoms by injecting polymethyl methacrylate into the fractured vertebral body (3, 4). New vertebral compression fractures (NVCFs) are one of the common complications following PVP (5). A meta-analysis (6) shows that 35.1% of patients experienced at least one vertebral re-fracture within 4 years of the initial fracture. The UK Clinical Guidelines on Osteoporosis Prevention and Treatment indicate that a history of previous fractures (particularly multiple vertebral fractures) can double the risk of fracture (7). The occurrence of OVCFs and NVCFs not only causes severe pain and functional impairment in patients (8), but also imposes a significant economic burden. In 2019, the total cost of osteoporotic fractures reported across European countries reached 56.9 billion euros, with re-fractures accounting for 20.6 billion euros (9).

Currently, most predictive models for postoperative NVCF following PVP focus on cement-related factors (such as leakage and distribution) or single bone density indicators, while neglecting the integrity of the skeletal and muscular systems. The pain and discomfort caused by OVCFs lead patients to reduce physical activity and exercise, resulting in degeneration of the paraspinal muscles (10). When patients lack sufficient physical activity, the capillary reactivity of the paraspinal muscles decreases, leading to inadequate blood supply and placing the muscles in a state of hypoxia (11). Persistent hypoxia results in insufficient glycogen utilization, triggering the accumulation of lactic acid and various metabolic byproducts, which in turn causes muscle edema and exacerbates back pain (12, 13). Over time, these effects cause the paraspinal muscles to gradually atrophy and degenerate, eventually being replaced by adipose tissue (14). Paraspinal muscle degeneration exerts a negative impact on the spine, leading to spinal instability and reducing the vertebrae’s ability to adapt to disturbances (such as trauma or falls), thereby providing a potential mechanism for the occurrence of NVCF (15). Early identification of paraspinal muscle degeneration and timely implementation of effective interventions to prevent NVCF can not only improve patient outcomes but also reduce the healthcare costs associated with re-fractures (16). Therefore, the early prevention and treatment of NVCF based on paraspinal muscle degeneration has become a key focus of clinical research.

Previous studies have shown (17–19) that gender, age, the distribution of fractured vertebrae, cement leakage, and bone CT value (Hounsfield unit—HU) are all independent risk factors for vertebral re-fracture. The occurrence of vertebral re-fracture is the result of the interaction of multiple factors (7) and is difficult to predict using a single indicator. Currently, research on predictive models for postoperative vertebral re-fractures following PVP is limited, and the factors included have not adequately accounted for the role of paraspinal muscles and vertebral bone density (20, 21). To address this gap, our study innovatively incorporated preoperative CT images to simultaneously quantify two key indicators—“vertebral bone density” and “paraspinal muscle area”—and integrated patients’ clinical characteristics, comorbidities, laboratory findings, and other baseline clinical data for the early prediction of NVCF risk. We aim to develop a comprehensive, easy-to-use, and visualized NVCF prediction model to assist clinicians in identifying high-risk patients at an early stage. Furthermore, this model will serve as a standardized tool for assessing rehabilitation prognosis after fracture, thereby facilitating further research in this field.

## 2 Materials and methods

### 2.1 Study designs and participants

We conducted a retrospective analysis and collected data from 1,112 patients with osteoporotic vertebral compression fractures (OVCFs) who were hospitalised in the Department of Orthopedics at Guangzhou University of Chinese Medicine’s Dongguan Hospital. Data were collected from January 1, 2022, to December 31, 2023. Inclusion criteria: (1) meeting the diagnostic criteria for OVCF established in the Chinese OrthopaedicAssociation (22) and confirmed by CT (determining the degree of vertebral compression, the integrity of the vertebral wall, and the presence of nerve compression) or MRI (identifying acute/new fractures through bone marrow edema signals, typically presenting as hypointensity on T1WI and hyperintensity on STIR or T2WI fat-suppression sequences). (2) Undergoing a CT scan within one week prior to surgery and receiving PVP surgical treatment. (3) Age ≥ 50 years. (4) Having a follow-up period of 12 to 24 months. Exclusion criteria: (1) lack of CT images or poor image quality. (2) Patients with baseline L4 fractures. (3) Patients with a history of vertebral fractures. (4) Patients with a history of spinal surgery. (5) Patients with severe comorbidities, including but not limited to malignant tumors, severe cardiovascular disease, and severe liver or kidney disease. (6) Patients with >10% missing data among the candidate predictors. (7) Patients with high-energy trauma (such as those from car accidents and falls from heights) and patients with fractures secondary to spinal tumors, infections, or other pathological factors.

A total of 423 patients with OVCF who underwent PVP were ultimately included. During follow-up, patients were diagnosed with NVCF if they developed low back pain or an acute decline in activities of daily living, and had corresponding imaging features. This study was conducted in accordance with the principles of the Declaration of Helsinki, and it was granted ethical permission by the Ethics Committee of Dongguan Hospital, Guangzhou University of Chinese Medicine(PJ[2026]No.189). Given that this retrospective observational study would not compromise patient privacy or safety, the Ethics Committee exempted the requirement for informed consent. To protect patient privacy, the data of this study will not be made public.

### 2.2 Clinical data acquisition

**Figure 1:**
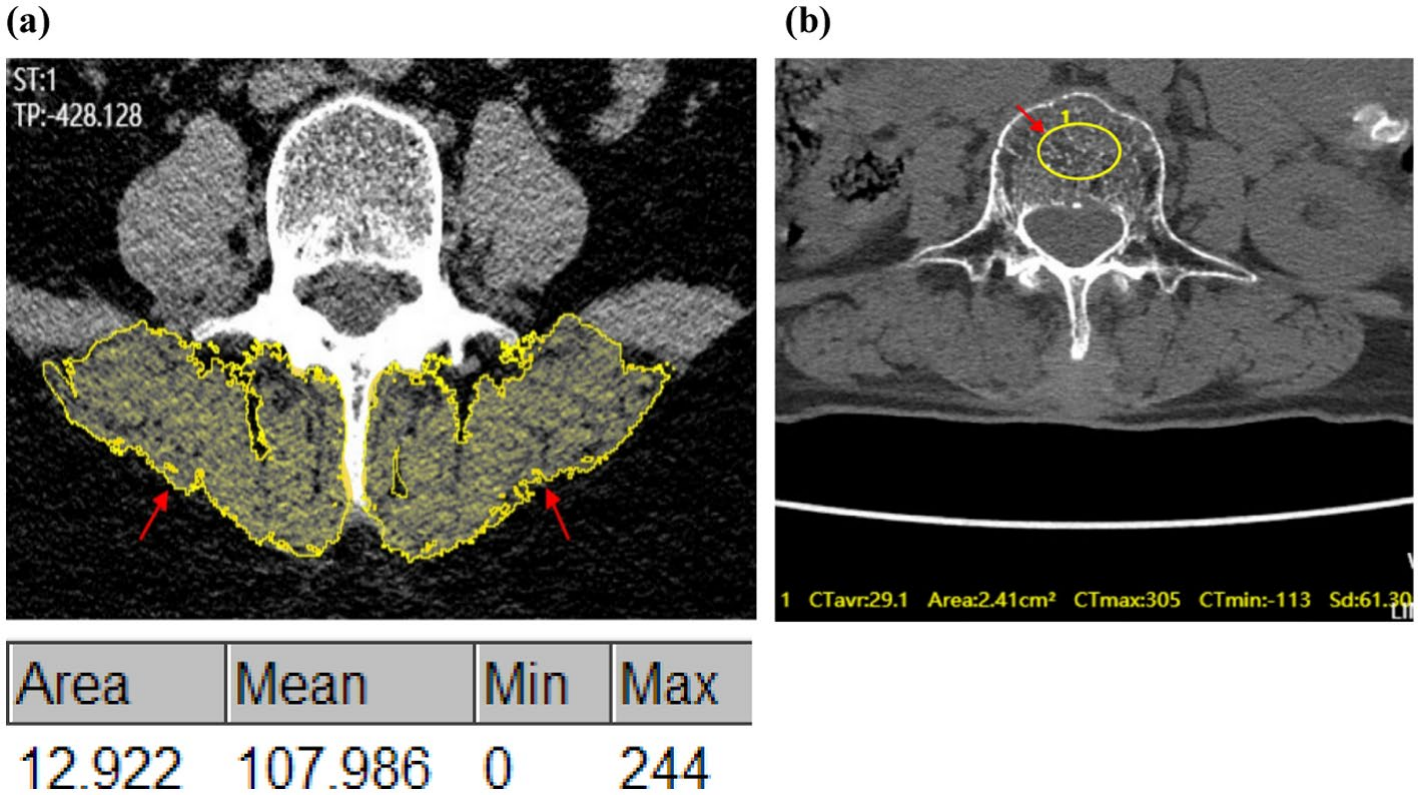
The measurements of muscle area **(A)** and bone value **(B)**. The bone value and the area of the paraspinal muscles on both sides were measured on axial CT images at the medium level of the L4 vertebrae.

Through literature review and clinical expertise, we identified 24 easily available clinical variables. Data were systematically extracted from patients’ electronic medical records, including: (1) Demographic information: gender, age, smoking history (smoking more than one cigarette per day and smoking continuously or cumulatively for 6 months or more) (23), alcohol consumption history (a history of long-term alcohol consumption, generally lasting more than 5 years, equivalent to ethanol intake of ≥40 g per day for men and ≥20 g per day for women) (24), medical history (chronic kidney disease, hypertension, diabetes), history of low-energy trauma, presence of bone cement leakage (based on the postoperative CT images, according to the classification criteria of Yeom et al. (25), it was classified as type B (via vertebral basilar vein), type S (via segmental vein), and type C (via bone cortical defect).), presence of single-segment fracture, fracture segment, cement volume and lumbar vertebrae T-value (T-L). (2) Laboratory parameters: creatinine (Cr), UREA, albumin (ALB), aspartate transaminase/alanine transaminase (AST/ALT), total cholesterol (TC), triglycerides (TG), high-density lipoprotein (HDL). (3) Paraspinal muscle area (cm^2^): Based on previous studies (26, 27), the fascial boundaries of the L4 paraspinal muscles (including bilateral erector spinae and multifidus muscles) were outlined using ImageJ, and the total cross-sectional area (CSA) on both sides was measured. Measurements were taken at three consecutive planes, and the average value was calculated.(4) Bone mass assessment: CT value of bone can reflect bone density (28). The CT scan parameters were as follows: voltage 120 kV, 240 mAs, slice thickness 1 mm. On axial CT images, an elliptical region of interest (ROI) measuring 2–3 cm^2^ is placed within the cancellous bone region of the mid-segment of the L4 vertebra to measure the CT value (Hounsfield Unit, HU). Lower bone CT value indicates lower bone density. According to previous studies (29), a vertebral CT value <135 HU is considered indicative of low bone mass. The measurement methods for paraspinal muscle area and bone mass are shown in Figure 1.

After excluding the patients with severely poor data integrity, we used the multiple imputation method in SPSS to fill in the missing values for the remaining patients in the included cohort, where the missing rate of each variable was less than 10%. Sensitivity analyses confirmed that the study results were robust to both missing data and imputation methods (Supplementary material 1).

### 2.3 Statistical analysis

Statistical analyses were primarily conducted using R software (version 4.4.3) and SPSS software (version 27.0). Descriptive statistical analyses were performed on 423 participants. (1) Continuous data that followed a normal distribution were expressed as mean ± standard deviation (SD). Categorical data were presented as frequency (%). For data that were not normally distributed, the median and interquartile range were used. Categorical variables were evaluated using the chi-square test, whilst continuous variables were analysed using the Mann–Whitney U test. (2) To ensure the fairness and comparability of the Lasso regression, all continuous predictor variables were standardised to Z-scores (mean = 0, standard deviation = 1) using the ‘scale’ function in R prior to model fitting. To assess the stability and generalisation ability of the model, optimal parameters were determined using 10-fold cross-validation. (3) Multicollinearity diagnostics were performed on all candidate predictor variables by calculating the Variance Inflation Factor (VIF), followed by variable selection using Lasso regression. To determine the optimal penalty coefficient, the lambda value was selected that fell within one standard deviation of the minimum cross-validation error. The variables selected by the Lasso method were subsequently incorporated into a multivariate logistic regression model, and multicollinearity was re-examined to ensure the stability of the parameter estimates. (4) The model’s discriminatory ability was assessed using Receiver Operating Characteristic (ROC) curves and the Area Under the Curve (AUC). The model’s goodness-of-fit and calibration were comprehensively assessed using the Hosmer-Lemeshow test and calibration curves. Furthermore, Decision Curve Analysis (DCA) and Clinical Impact Curve (CIC) were employed to evaluate the model’s clinical net benefit at different decision thresholds, thereby exploring its clinical utility.

## 3 Results

### 3.1 Patient characteristics

**Figure 2:**
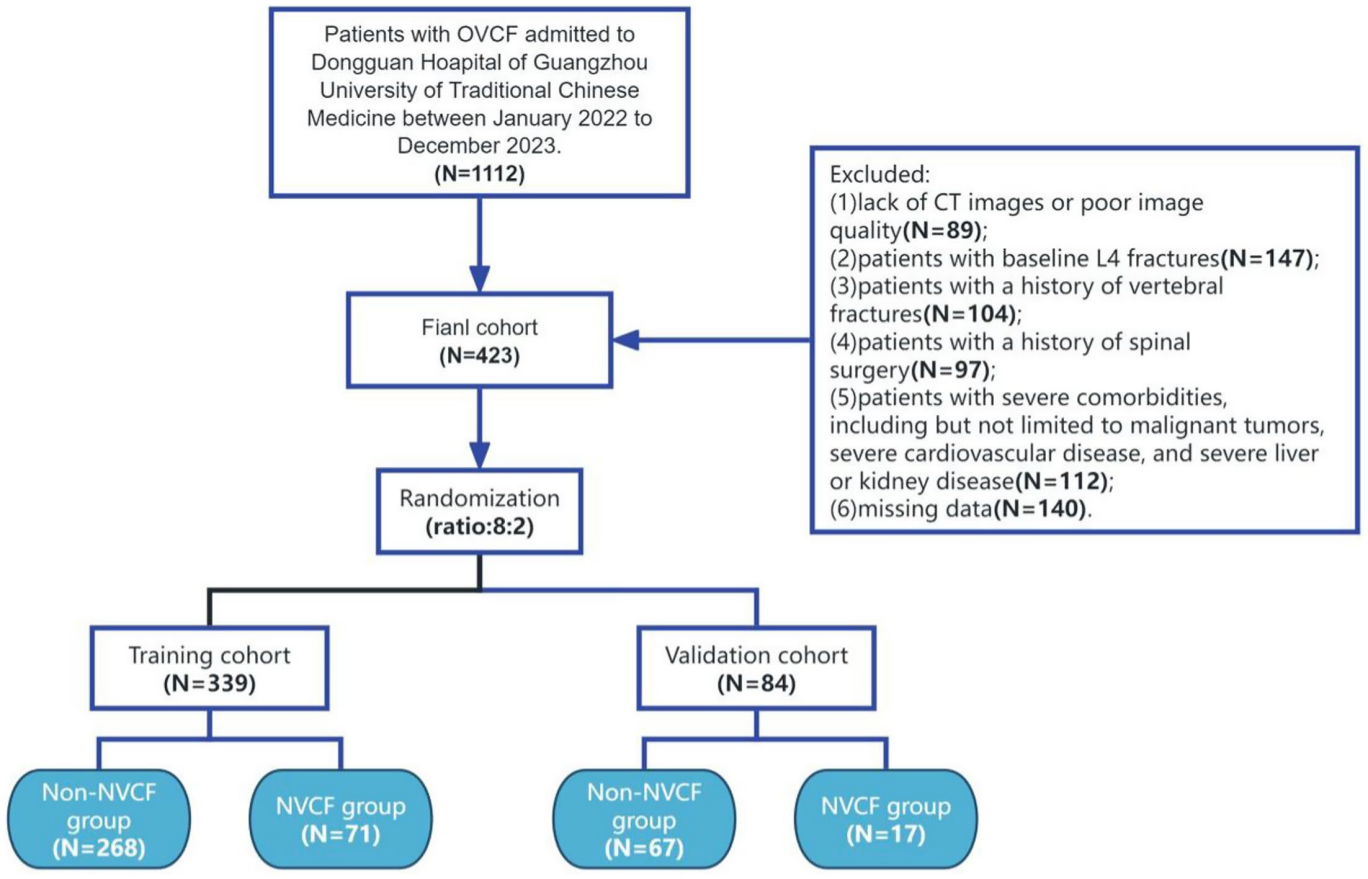
Flowchart.

Of the 1,112 patients with OVCF undergoing routine PVP surgery included in this study, 689 did not meet the inclusion criteria. Ultimately, 88 (20.8%) of the 423 study participants were diagnosed with NVCF. Following an 8:2 random sampling ratio, 339 cases were allocated to the training set and 84 cases to the validation set. The flowchart was shown in Figure 2. Furthermore, Table 1 presented the differences between the NVCF group and the non-NVCF group in terms of demographic data, clinical characteristics, relevant indices and laboratory parameters.

**Table 1:** Baseline characteristics of the NVCF and Non-NVCF groups.

Variable	Option	Total (*n* = 423)	Refracture (*n* = 88)	Non_refracture (*n* = 335)	*P*-value
Demographic					
Age (years, mean±SD)		72.95 ± 9.56	78.53 ± 8.35	71.48 ± 9.33	**<0.001**
Gender (*n*, %)	Male	365 (86.3%)	79 (89.8%)	286 (85.4%)	0.286
	Female	58 (13.7%)	9 (10.2%)	49 (14.6%)	
Medical history					
Low energy trauma (*n*, %)	No	162 (38.3%)	55 (62.5%)	107 (31.9%)	**<0.001**
	Yes	261 (61.7%)	33 (37.5%)	228 (68.1%)	
Smoke (*n*, %)	No	417 (98.6%)	88 (100%)	329 (98.2%)	0.206
	Yes	6 (1.4%)	0 (0%)	6 (1.8%)	
Alcohol (*n*, %)	No	420 (99.3%)	88 (100%)	332 (99.1%)	0.373
	Yes	3 (0.7%)	0 (0%)	3 (0.9%)	
Chronic kidney disease (*n*, %)	No	417 (98.6%)	87 (98.9%)	330 (98.5%)	0.801
	Yes	6 (1.4%)	1 (1.1%)	5 (1.5%)	
Diabetes (*n*, %)	No	364 (86.1%)	81 (92.0%)	283 (84.5%)	0.068
	Yes	59 (13.9%)	7 (8.0%)	52 (15.5%)	
Hypertension (*n*, %)	No	245 (57.9%)	45 (51.1%)	200 (59.7%)	0.148
	Yes	178 (42.1%)	43 (48.9%)	135 (40.3%)	
Bone cement leaking (*n*, %)	No	400 (94.6%)	87 (98.9%)	313 (93.4%)	**0.046**
	Yes	23 (5.4%)	1 (1.1%)	22 (6.6%)	
Clinical features					
Single segment (*n*, %)	No	63 (14.9%)	24 (27.3%)	39 (11.6%)	**<0.001**
	Yes	360 (85.1%)	64 (72.7%)	296 (88.4%)	
Bone cement volume (mean±SD)		6.61 ± 2.62	6.96 ± 3.29	6.52 ± 2.41	0.226
T11-L2 Fracture (*n*, %)	No	95 (22.5%)	28 (31.8%)	67 (20.0%)	**0.018**
	Yes	328 (77.5%)	60 (68.2%)	268 (80.0%)	
Inspection results					
Cr (mean±SD)		68.30 ± 22.00	67.51 ± 17.28	68.51 ± 23.10	0.855
UREA (mean±SD)		5.91 ± 2.12	5.76 ± 1.78	5.95 ± 2.21	0.758
ALB (mean±SD)		38.77 ± 3.45	38.53 ± 3.70	38.83 ± 3.39	0.482
AST/ALT (mean±SD)		1.36 ± 0.60	1.45 ± 0.60	1.34 ± 0.60	**0.02**
TC (mean±SD)		4.77 ± 1.01	4.92 ± 1.04	4.73 ± 1.00	0.187
TG (mean±SD)		1.26 ± 0.68	1.08 ± 0.35	1.30 ± 0.73	0.071
HDL (mean±SD)		1.31 ± 0.33	1.41 ± 0.35	1.29 ± 0.32	**0.011**
Paravertebral muscle area (mean±SD)		11.77 ± 3.23	9.22 ± 2.80	12.33 ± 3.05	**<0.001**
Lumbar vertebra T value (mean±SD)		−3.06 ± 1.24	−3.62 ± 1.29	−2.94 ± 1.20	**<0.001**
Bone CT value (mean±SD)		58.69 ± 42.27	14.39 ± 30.62	68.17 ± 38.21	**<0.001**

Bold indicates *p* < 0.05.

Cr, creatinine; ALB, albumin; AST/ALT, aspartate transaminase/alanine transaminase; TC, total cholesterol; TG, triglycerides; HDL, high-density lipoprotein; T-L, *T*-value of lumbar vertebrae.

### 3.2 Selection of predictive factors

**Figure 3:**
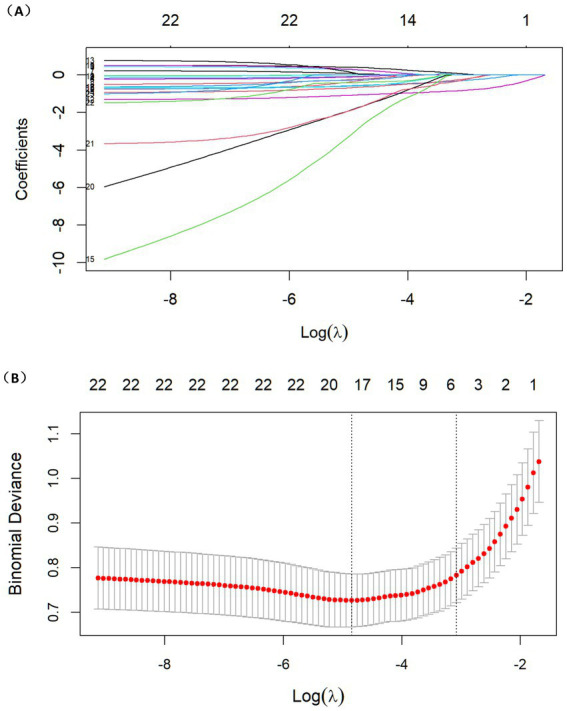
LASSO Regression. **(A)** Lasso regression coefficient distribution curve. **(B)** The optimal parameter (lambda) selection in the model employed ten-fold cross-validation using a minimisation criterion. Vertical dashed lines are drawn to, respectively, indicate lambda.min and lambda.1se (the one standard error criterion) to determine the optimal predictive variables.

Multicollinearity analysis was performed for all variables (Supplementary Table 1). Lasso regression was used to screen for predictive factors, and the optimal coefficient *λ* was determined using 10-fold cross-validation and a minimisation criterion, finally identifying 10 predictive variables with non-zero coefficients, including age, smoking history, diabetes, paraspinal muscle area, bone CT value, albumin (ALB), non-thoracolumbar fractures, presence of bone cement leakage, history of low-energy trauma and single-segment fracture (Figures 3A,B). We then performed a multivariate logistic regression analysis on the predictors identified by Lasso regression. The results showed that age (OR: 1.803, 95%CI: 1.21–2.687), ALB (OR: 1.584, 95%CI: 1.056–2.377), bone CT value (OR:0.33, 95%CI: 0.181–0.600), paraspinal muscle area (OR:0.524, 95%CI: 0.324–0.849), low-energy trauma (OR: 0.453, 95%CI: 0.234–0.878) and single-segment fracture (OR: 0.311, 95%CI: 0.141–0.686) were confirmed as independent factors influencing postoperative vertebral re-fracture following PVP (Table 2). The variance inflation factor (VIF) values for all predictors were significantly below the threshold of 5, indicating the absence of severe multicollinearity (Supplementary Table 2).

**Table 2:** Multivariable logistic regression results for predicting NVCF patients.

Variable	OR	95%CI	p-value
Age	1.803	1.210–2.687	0.004
ALB	1.584	1.056–2.377	0.029
Paravertebral muscle area	0.524	0.324–0.849	0.012
Bone CT value	0.330	0.181–0.600	0.001
Low energy trauma	0.453	0.234–0.878	0.02
Single segment	0.311	0.141–0.686	0.004
Diabetes	0.309	0.086–1.110	0.073
Bone cement leaking	0	0-Inf	0.990
Smoke	0	0-Inf	0.994

OR, odds ratio; Cl, confidence interval.

### 3.3 Construction of the nomogram

**Figure 4:**
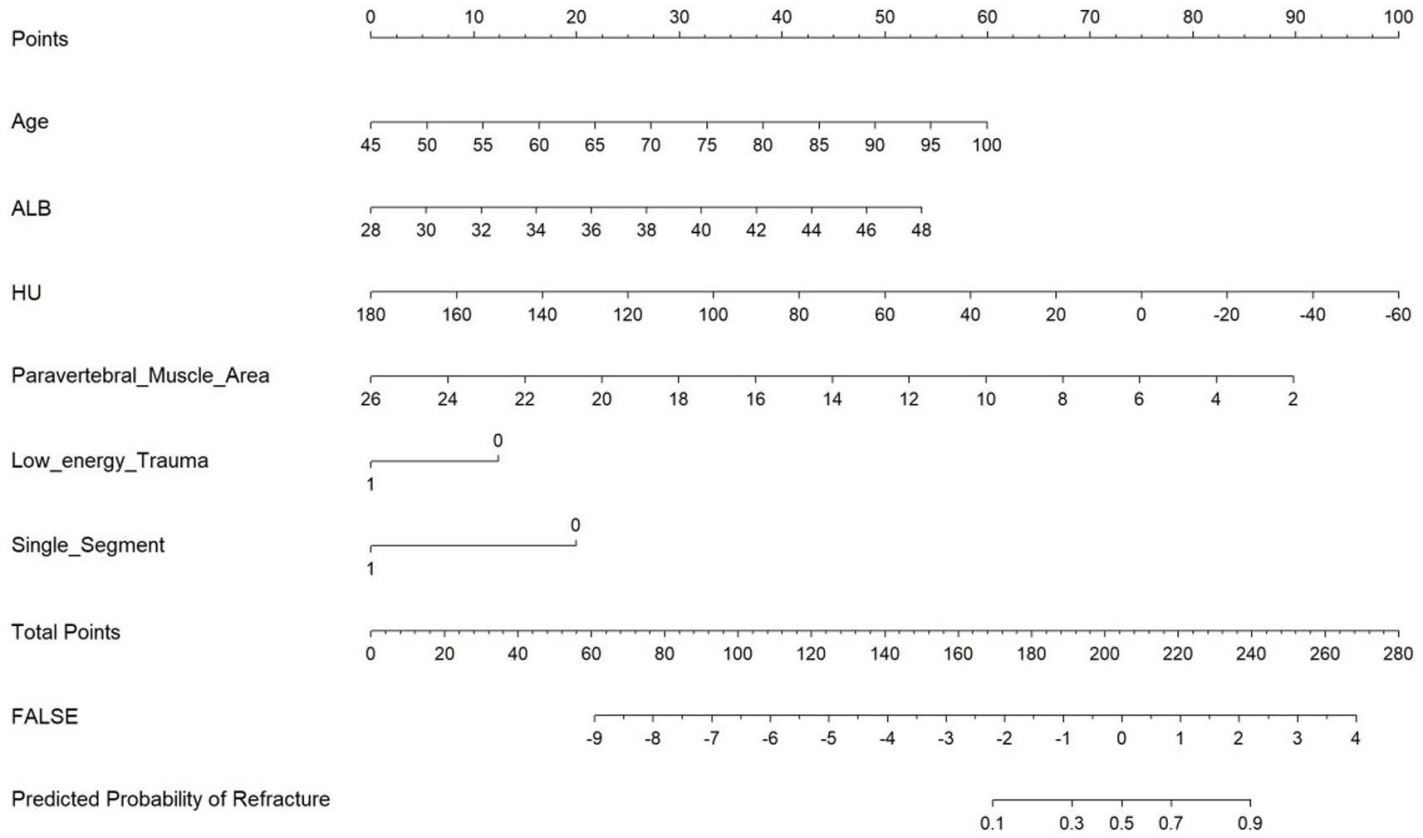
Nomogram prediction model for patients with NVCF.

**Figure 5:**
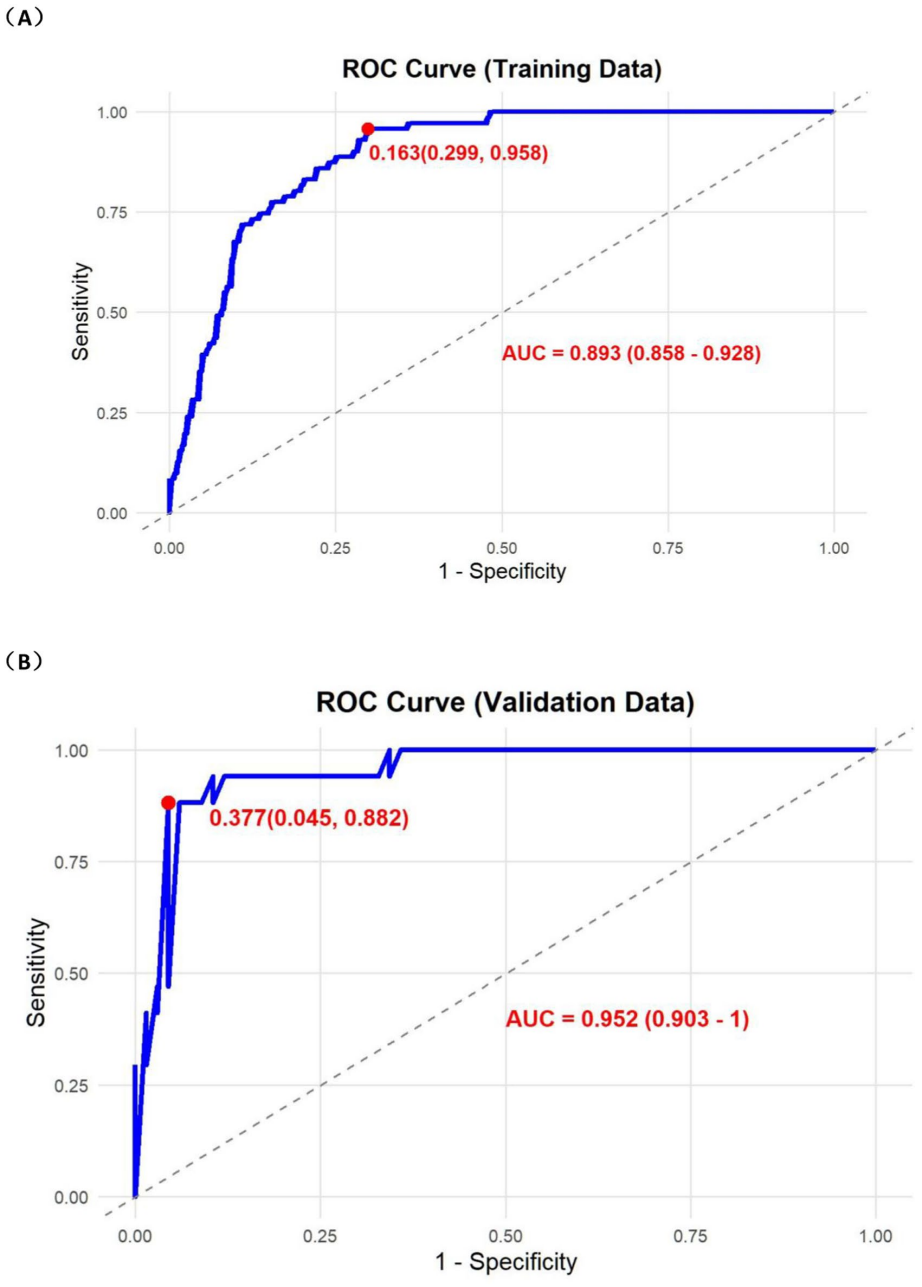
ROC curves for the model in both the training **(A)** and validation **(B)** sets.

Based on the results of multivariate logistic regression, we constructed a nomogram for patients with NVCF (Figure 4). The Area Under the Curve (AUC) for the training set and validation set were, respectively, 0.893 (95%CI: 0.858–0.928) and 0.952 (95%CI: 0.903–1), indicating that the model possesses high predictive accuracy and good discriminatory power (Figure 5). The optimal discrimination threshold was determined by maximising the Youden’s index. In the training set, a Youden index of 0.6592 corresponded to an optimal cut-off value of 0.1628, at which point the sensitivity was 95.77% and the specificity was 70.15%. In the validation set, a Youden index of 0.8376 corresponds to an optimal cut-off value of 0.3766, yielded a sensitivity of 88.24% and a specificity of 95.52%. These results demonstrated that the nomogram exhibits stable discriminatory performance and has broad prospects for clinical application. Furthermore, high negative predictive values (training set 85.91%, validation set 87.67%) indicated that the model is reliable for diagnostic exclusion. The comprehensive performance metrics were summarised in Table 3.

**Table 3:** Diagnostic performance indicators of the model based on preset clinical decision thresholds.

Set	Cut-off value	Sensitivity	Specificity	PPV	NPV	AUC (95%CI)
Training set	0.1628	0.9577	0.7015	0.625	0.8591	0.893(0.858–0.928)
Validation set	0.3766	0.8824	0.9552	0.7273	0.8767	0.924(0.903–1.000)

### 3.4 Internal validation strategy

This study employed bootstrap for internal validation: After 1,000 rounds of bootstrap resampling for optimism correction, the model achieved a C-index of 0.881. The result indicated that the model provided a reliable means of performance evaluation.

### 3.5 Validation of the nomogram

**Figure 6:**
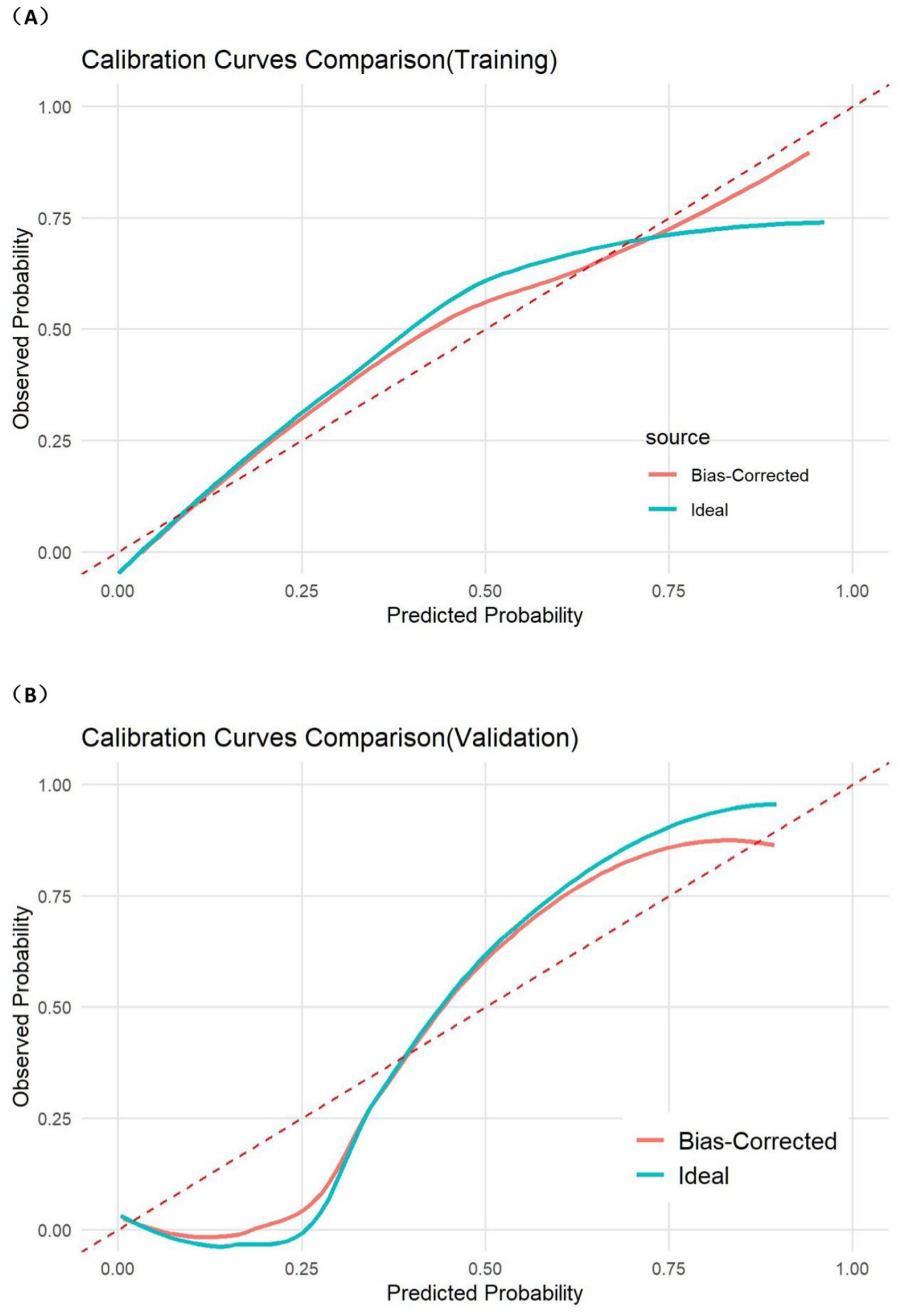
Calibration curves for the NVCF nomogram prediction **(A, B)**. The diagonal dashed line signifies perfect prediction by the optimal model. The proximity of the green solid line to this dashed line indicates the model’s predictive performance; the closer they are, the more accurate the predictions.

This study employed the Hosmer-Lemeshow test to assess the goodness-of-fit. The calibration curves (Figures 6A,B) demonstrate a strong correlation between predicted probabilities and observed outcomes in both the training and validation sets. However, the calibration curve for the validation set reveals a slight systematic bias: risk is overestimated in the low-probability range and underestimated in the high-probability range.

**Figure 7:**
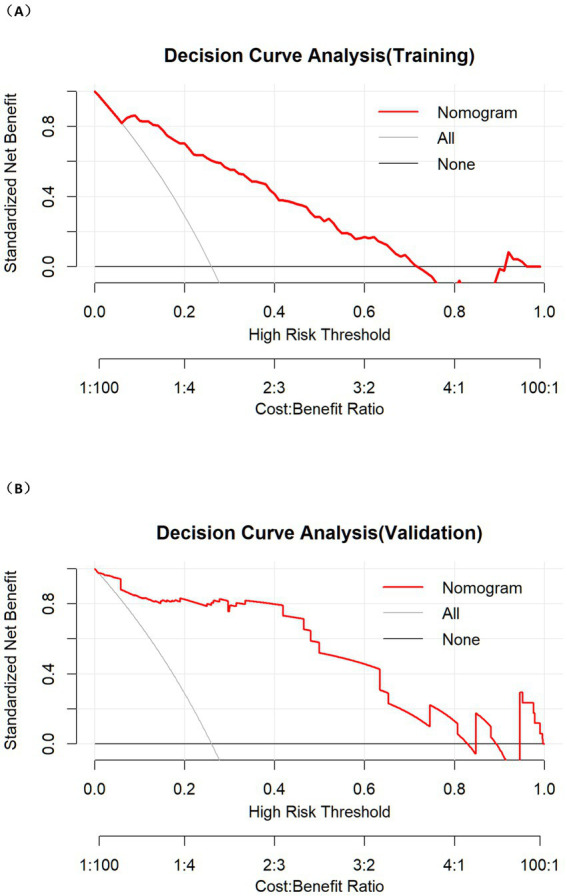
DCA for the NVCF nomogram prediction **(A, B)**. The y-axis measures the net benefit obtained from using the model. The red line in the figure represents the clinical diagnostic model for NVCF. In contrast, the black horizontal line (None line) and the gray diagonal line (All line) represent the extreme cases of “no intervention” and “all intervention,” respectively.

We further employed Decision Curve Analysis (DCA) to evaluate the clinical utility of this nomogram. As shown in Figure 7, the DCA curves show that the model offers a net clinical benefit for patients within a reasonable range of probability thresholds, in both the training set and the validation set. In the training set (a), when the range of probability thresholds lies between approximately 5 and 75%, and in the validation set (b), when the range of probability thresholds lies between approximately 3 and 83%, the standardised net benefits of the nomogram all exceeded those of the extreme strategies (treat all or treat none), suggesting its broad clinical applicability.

**Figure 8:**
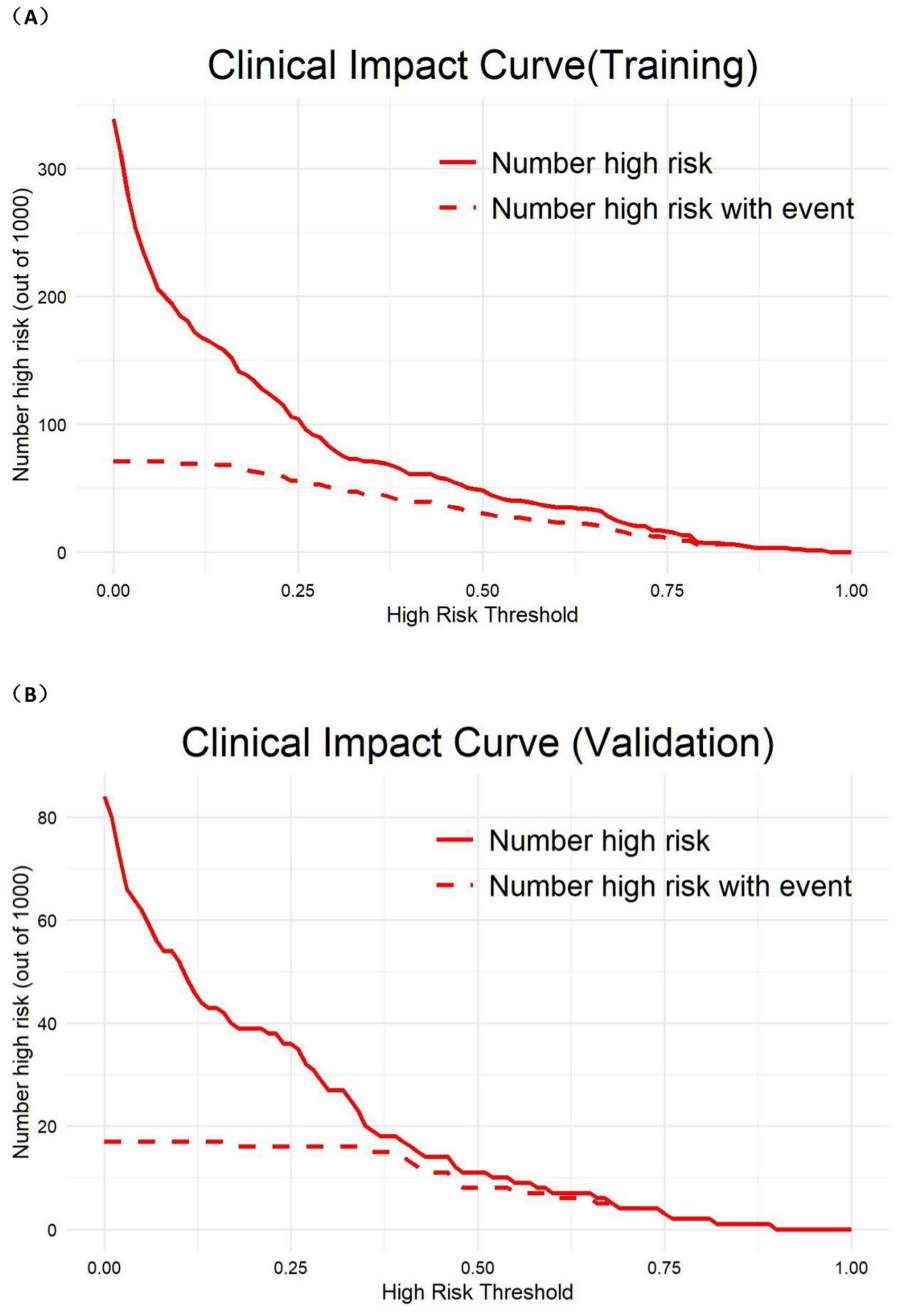
CIC for the NVCF nomogram prediction **(A, B)**. The y-axis represents the number of individuals at risk. The red line indicates the predicted number of events occurring for NVCF, whereas the red dashed line depicts the actual occurrences.

CIC analysis (Figures 8A,B) confirms that the model possesses excellent calibration capabilities. In particular, when the high-risk threshold is set at about 65% or higher, the predicted number of positive events shows a high degree of consistency with the actual incidence rate. It indicates that the model exhibits superior calibration performance within this range, effectively minimising the occurrence of over-prediction (false positives). Consequently, when clinicians use this threshold to screen high-risk populations, they can not only accurately identify patients requiring intervention but also maintain a low number needed to treat (NNT), fully demonstrating the model’s high reliability and clinical utility.

## 4 Discussion

Paraspinal muscles play a critical role in maintaining the biomechanical stability of the spine (30), and their degeneration can lead to changes in spinal morphology, impair spinal function, and be closely associated with various spinal disorders (31). This study shows that the incidence of NVCF following PVP surgery is 20.8%, and paraspinal muscle atrophy is closely associated with the occurrence of NVCF. However, current clinical studies on NVCF have primarily focused on factors such as spinal morphology and bone cement (32), neglecting the role of paraspinal muscles. In recent years, nomograms, as visual statistical tool that integrates key parameters to predict disease occurrence, have been widely adopted in the clinical field (33). This tool assists clinicians in making rapid and convenient predictions and assessments by translating complex mathematical models into intuitive graphics. This study developed and internally validated a prediction model based on a nomogram to assess the risk of NVCF in patients following PVP surgery, thereby enabling the accurate identification of high-risk patients and the implementation of early intervention. The results indicate that age, albumin (ALB), paraspinal muscle area, bone CT value, low-energy trauma and single-segment fractures are key predictors of NVCF. Furthermore, the model demonstrates good predictive ability, excellent calibration performance, and high clinical utility.

Among patients with NVCF, advanced age is considered an independent risk factor associated with disease severity, prognosis, and the risk of complications (34), and older adults are more prone to developing NVCF. This may be related to physiological changes in skeletal structure and the endocrine system that occur during the aging process. Russell SJ (35) and colleagues proposed that reduced steroid secretion in the elderly, accompanied by increased oxidative stress, exacerbate bone loss. In addition, reduced physical activity among older adults further exacerbates osteoporosis and increases the likelihood of subsequent fractures.

Our multivariate logistic regression analysis unexpectedly revealed that higher serum albumin levels (OR: 1.584) are an independent risk factor for NVCF, associated with an increased risk. It appears to contradict conventional wisdom but may be related to the physiological complexity of albumin concentration. Previous studies have generally regarded low serum albumin as a marker of malnutrition in patients and as a result of inflammatory responses (36). However, a recent large-scale prospective study (37) revealed a unique U-shaped relationship between ALB and neurological recovery. This study indicated that serum albumin levels do not follow the traditional “the higher, the better” principle; when ALB levels exceed a certain threshold, they are associated with adverse outcomes. The underlying mechanisms may be related to the following two factors: First, during the acute stage of fracture or after severe trauma, the body is in a state of high metabolic stress, and skeletal muscle proteins are extensively broken down to provide energy. The amino acids produced from this breakdown are converted into glucose through gluconeogenesis in the liver, and at the same time, albumin is synthesized. The high level of albumin measured at this time actually reflects the extensive consumption of muscle proteins. This indicates that the patient may have significant muscle mass loss, decreased muscle strength and buffering capacity, and is unable to effectively protect the spine, thereby increasing the risk of recurrent fractures. Second, the blood supply to the vertebral body relies on a rich microcirculatory network. Excessively high albumin levels increase blood viscosity, reduce microcirculatory perfusion within the vertebral body, leading to insufficient microcirculatory perfusion within the bone, hindering bone repair, and increasing the risk of vertebral re-fracture. Therefore, the elevated albumin levels observed in this study may be situated on the ascending right-hand side of this U-shaped curve. It suggests that when assessing the risk of NVCF in clinical practice, we must not only be vigilant about the nutritional risks associated with hypoalbuminemia, but also pay attention to the hemodynamic and spinal biomechanical changes that hyperalbuminemia may cause. It is noteworthy that although the baseline comparison revealed no significant difference in ALB levels between the NVCF and non-NVCF groups (38.53 ± 3.70 vs. 38.83 ± 3.39, *p* = 0.482), ALB remained an independent predictor of NVCF after adjustment for confounders (OR = 1.584, *p* = 0.029). This seemingly paradoxical finding is actually attributable to a negative masking effect of confounding factors. Patients with NVCF frequently present with a systemic inflammatory state, and the inflammatory response itself can significantly consume or suppress ALB synthesis, thereby‘levelling off’ the mean ALB difference between the two groups at the univariate level. After removing these interferences in the multivariable model, the independent net effect of ALB emerged. This underscores that, in clinical prediction modelling, predictive variables should not be selected solely on the basis of univariate intergroup comparison *p*-values; rather, the adjusted independent effects derived from multivariable analysis are of greater reference value. Moreover, collinearity diagnostics (VIF = 1.674) ruled out multicollinearity, further supporting the robustness of our findings.

Paraspinal muscle degeneration is a complex and dynamic process that has profound negative effects on the spine. Previous studies (38, 39) have shown that paraspinal muscle degeneration leads to structural changes in the spine, impairs spinal function and exacerbates various spinal disorders. This study further confirms the association between paraspinal muscle atrophy and spinal disorders. First, under normal conditions, healthy paraspinal muscles bear the majority of the spinal compressive load. By contracting prior to limb movement, paraspinal muscles distribute and transmits axial loads along the spine. However, a reduction in paraspinal muscle area can directly lead to local sagittal imbalance (excessive segmental kyphosis) (40). Unlike global coronal deformity (scoliosis), which affects overall trunk balance, this local sagittal malalignment imposes more direct biomechanical stress on the adjacent vertebral bodies. When the segmental kyphotic angle exceeds 11°, the risk of vertebral re-fracture increases significantly (41). Second, degeneration of the paraspinal muscles involves not only muscle atrophy but is also accompanied by fatty infiltration (42). The paraspinal muscles are rich in muscle spindles, which serve as an important source of spinal proprioception (43). When fat content in the muscles increases, proprioceptive signals are disrupted, and feedforward activation may be delayed, leading to compensatory activation of superficial muscles and increased mechanical stress on the spine (44), thereby compromising spinal stability. Furthermore, atrophied muscle tissue releases pro-inflammatory cytokines, which increase osteoclast production, accelerate local bone resorption and affect bone metabolism in adjacent vertebrae (45), creating a specific pathological state that accelerates the onset of NVCF. Based on this, it is hypothesized that incorporating the assessment of paraspinal muscle mass into the standard clinical pathway for post-PVP rehabilitation may be of critical importance in preventing NVCF.

Although all participants included in this study met the diagnostic criteria for OVCF and had already reached a low bone mass level, the results indicate that even within the established pathological category of osteoporosis, bone CT value still continues to perform the risk stratification function as a continuous variable. The underlying mechanism may lie in the fact that PVP surgery significantly alters the mechanical conduction mode of the vertebral body. The stiffness of bone cement is 7–10 times that of osteoporotic cancellous bone (46). The lower the bone CT value, the more fragile the trabecular bone becomes. This results in a significant disparity in elastic modulus between the vertebral body and the bone cement, leading to stress concentration. Consequently, the load cannot be uniformly transmitted through the trabecular bone but is transmitted via the rigid bone cement column to the endplate or adjacent vertebral body. Furthermore, low bone mass is often accompanied by widened trabecular spaces (47). While this facilitates the diffusion and filling of bone cement, it increases the risk of bone cement leakage into the intervertebral disc or the posterior aspect of the vertebral body. Bone cement leakage alters the mechanical stiffness of the intervertebral disc and weakens the endplates of adjacent vertebrae (48), thereby increasing the risk of degeneration and fracture in adjacent segments. It offers a new perspective on postoperative rehabilitation following PVP. Patients should not be diagnosed with osteoporosis in a generalized manner; instead, preoperative CT scan should be performed to measure bone CT value. This approach is not only simple, low-cost, and quantifiable but also helps clinicians identify high-risk patients who may develop NVCFs. For patients with extremely low bone CT value, stricter postoperative treatment with antiosteoporotic medications is necessary to prevent or reduce the incidence of NVCFs.

Our study indicates that low-energy trauma is a protective factor for NVCF (OR = 0.453). It means that compared to the group without low-energy trauma (the “spontaneous fracture” group), patients with a clear history of low-energy trauma have a relatively lower risk of re-fracture. To distinguish from “spontaneous fractures” (fractures that occur without a clear history of trauma or that happen only under normal physiological loads, such as coughing and bending over), in this study, “low-energy trauma” is clearly defined as a fall from a standing position at a considerable height or a minor external force impact (excluding high-energy mechanisms such as motor vehicle collisions or falls from heights). And all the enrolled cases were excluded from having confirmed spinal malignant tumors or infectious lesions through imaging or clinical examinations. After this rigorous screening, low-energy trauma was found to be a protective factor. However, this protective effect should be interpreted as a surrogate marker for the spinal loading status, rather than a biomechanical causal protection. This means that compared to patients who have been bedridden for a long time or have no history of trauma, those with a clear history of low-energy trauma have the ability to perform basic activities, and thus their vertebrae are subjected to regular repetitive stress. This, statistically, results in a lower incidence of re-fracture. The research conducted by Brennan et al. (49) indicates that a history of previous immobility supports the diagnosis of spontaneous fractures; Paola Pisani et al. (50) also found in their study that immobility is a significant cause of bone loss. Women who were immobilized for one month experienced more bone loss during the process of osteoporosis compared to normal cases over one or two years (51). Relatively speaking, patients with a clear history of low-energy trauma tend to have better mobility, and their vertebrae are subjected to greater repetitive stress and instantaneous impact. Moreover, this study focuses on the role of the cross-sectional area of paravertebral muscles, and muscle mass is closely related to mobility. Therefore, low-energy trauma should be regarded as a substitute indicator for low spinal load status and restrictive activities due to frailty, rather than an independent biomechanical protective factor.

Our study unexpectedly found that single-segment fracture acts as a protective factor against NVCF. The paraspinal muscles constitute the active system responsible for maintaining the stability of the neutral zone of the spine. Among them, the multifidus muscles, as the deep stabilizing muscles, play a crucial role in maintaining the control of spinal segments (52). The paraspinal muscle injuries and degeneration in patients with single-segment fractures are relatively mild. The muscles can absorb and buffer the spinal vibration load, reducing abnormal spinal displacement and thereby protecting other vertebrae from abnormal stress impacts. Additionally, according to Wolff’s law (53), the mechanical stress generated by the muscles attached to the bones during exertion can stimulate bone cells, promote osteoblast differentiation and inhibit osteoclast activity, thereby maintaining bone density. Patients with single-segment fractures retain more functional muscle fibers and a larger muscle cross-sectional area, which enables them to maintain normal mechanical transmission, slow down the rate of bone density loss, and prevent the occurrence of re-fractures.

The most significant strength of our model lies in its clinical accessibility. Unlike previous studies that primarily relied on MRI for soft tissue assessment (54–56), our study presents a comprehensive evaluation strategy based on CT imaging. CT offers superior accessibility and rapidity in clinical trauma settings. By integrating bone quality (Vertebral CT Attenuation Value) and muscle parameters (paraspinal muscle area) into a single preoperative workflow, we eliminated the need for additional MRI scans, thereby significantly reducing medical costs and streamlining the preoperative decision-making process, allowing surgeons to rapidly calculate the risk probability at the point of care without incurring additional economic costs or delaying treatment decisions. Furthermore, distinct from studies (57, 58) focusing solely on radiological parameters, we employed a multidimensional assessment approach. Using LASSO regression, our model integrates readily available risk factors, encompassing demographics (age), nutritional status (ALB), morphological parameters (paraspinal muscle area, bone CT value), and injury characteristics (low-energy trauma, single-segment fracture). This multidimensional model, combining clinical and imaging features, likely provides more comprehensive prognostic information than unidimensional assessments. Finally, focusing on the short-term risk (1–2 years) is clinically vital, as this period represents a critical window for secondary fracture prevention. Our model serves as an effective early warning system. By identifying patients at high risk of refracture immediately after the index surgery, clinicians can shift from a reactive approach to a proactive one. This is particularly important given that refractures often lead to a vicious cycle of immobility, further bone loss, and increased mortality.

In addition, although we have validated the predictive value of CT-based bone mineral density values and muscle area, it is important to acknowledge the evolving landscape of bone quality assessment. Emerging MRI-based biomarkers, such as the Vertebral Bone Quality (VBQ) score, have recently been validated as effective predictors of bone density and osteoporosis in spinal surgery patients (59). Although our current model relies on CT imaging, future prospective studies could explore the integration of the VBQ score (derived from routine pre-operative MRI) with our CT-based metrics. Such a multimodal approach may offer a more comprehensive evaluation of bone quality and further improve risk stratification.

## 5 Conclusion

Our study found that age, albumin levels, paraspinal muscle area, bone CT value, low-energy trauma and single-segment fracture were significantly associated with the incidence of NVCF following PVP surgery. Based on these findings, a practical predictive nomogram was developed. The core advantage of this model lies in its ability to accurately predict the recurrence of NVCF following PVP surgery based on routine clinical data, offering both cost-effectiveness and ease-of-use. To validate the effectiveness of this nomogram model, future large-scale, multicenter, prospective studies are needed to obtain comprehensive data. Until reliable external validation evidence is obtained, this model should not be used directly as a basis for clinical treatment decisions.

## 6 Limitations

This study has several limitations. First, as a retrospective single-center investigation with a limited sample size, it is inevitably subject to missing data and selection bias. For instance, not all important risk factors for fractures (such as body mass index) were included, and patients with fractures at the L4 vertebra level were excluded. Moreover, we excluded patients with a history of vertebral fractures, so the conclusions are primarily applicable to the population with first-time OVCF; extrapolation to patients with recurrent or multilevel old fractures should be undertaken with caution. Second, the model exhibited calibration bias in the validation set (overestimation in the low-risk interval and underestimation in the high-risk interval), which may be attributable to differences in baseline characteristics between the training and validation cohorts or to the relatively small sample size of the validation set. Future work should therefore involve large-scale, multicenter, prospective external validation, combined with post-processing calibration techniques (e.g., Platt scaling or isotonic regression) to further enhance model performance. Nevertheless, the model maintained a high negative predictive value (NPV = 87.67%), underscoring its practical value as a clinical screening tool—it can effectively rule out low-risk patients and direct high-risk individuals to undergo gold-standard confirmatory testing, thereby minimizing missed diagnoses. In addition, this study may not have encompassed all potential etiologies of postoperative NVCF, and the absence of prospective data implies that other important indicators might also serve as candidate predictors, which warrants further exploration. Furthermore, we only evaluated the cross-sectional area of skeletal muscle without quantitatively measuring CT attenuation values to reflect intramuscular fat infiltration; a multi-index combined analysis incorporating both muscle area and CT density in future studies would allow a more comprehensive assessment of muscle degeneration. Lastly, due to constraints inherent in the original data structure, subgroup analyses based on gender or body type could not be conducted.

## Data Availability

The original contributions presented in the study are included in the article/Supplementary material, further inquiries can be directed to the corresponding author/s.
