# Supplementary material for: Development and validation of a CT-based predictive model for new vertebral compression fractures: the role of vertebral CT attenuation and paraspinal muscle
Source: Front Med (Lausanne). 2026 Jul 22;13:1860827. doi: 10.3389/fmed.2026.1860827 (PMC13437277; doi:10.3389/fmed.2026.1860827)
Supplement: Supplementary file 1 [file Data_Sheet_1.docx]

**Development and Validation of a CT-Based Predictive Model for New Vertebral Compression Fractures: The Role of Vertebral BMD and Paraspinal Muscle**

**Yiqi Wu^1^，Qing Song^1^，Canwei Hu^1^，Chaoyi Yin^1^，Zhiqing Chen^1^，Pujian Zhong^1^，Binshan Zhang^1*^**

**^1^**Guangzhou University of Traditional Chinese Medicine Dongguan Hospital, China

*** Correspondence:**
Binshan Zhang

13600298950@163.com

### Supplementary Material 1:Handling of Missing Data

1. **Assessment of Missing Data**

Prior to analysis, we assessed the pattern and extent of missingness in the candidate predictor variables among the initial study cohort. Only **eight** variables were found to have missing values: Bone Cement Volume was missing in 1 cases (0.29%), Creatinine（Cr）in 2 cases (0.59%), UREA in 2 cases (0.59%), Albumin (ALB) in 12 cases (3.54%), AST/ALT in 2 cases (0.59%), Paravertebral_Muscle_Area in 15 cases (4.42%), Lumbar Vertebra T Value in 35 cases (8.27%) and Bone CT Value in 18 cases (5.31%). The overall missing data rate for each variable was below 10%, which is considered acceptable for employing multiple imputation techniques rather than complete-case analysis, thereby preserving statistical power and reducing potential bias ^[1-2]^.

2. **Imputation Strategy**

To handle the missing data, we employed Multiple Imputation (MI) using the R software (version 4.4.3). The imputation process was based on the Fully Conditional Specification (FCS) algorithm, also known as Multiple Imputation by Chained Equations (MICE). This method iteratively imputes missing values for each variable conditional on the other variables in the dataset, which is particularly suitable for mixed data types (continuous and categorical). We generated 6 imputed datasets over 1,000 iterations. The results from these datasets were then pooled for the final analysis according to Rubin's rules, which appropriately reflects the uncertainty introduced by the missing data .

3. **Sensitivity Analysis for Robustness**

To rigorously evaluate the impact of the missing data and the imputation method on our primary findings, we conducted a sensitivity analysis by constructing a "complete-case dataset." This dataset was created by excluding 1 cases missing Bone Cement Volume, 2 cases missing Creatinine（Cr）, 2 cases missing UREA, 12 cases missing Albumin (ALB), 2 cases missing AST/ALT, 15 cases missing Paravertebral Muscle Area, 35 cases missing Lumbar Vertebra T Value and 18 cases missing Bone CT Value. The primary prediction model was then rebuilt using this dataset.

4. **Results of Sensitivity Analysis**

The performance of the model derived from the imputed data was compared with that from the complete-case dataset. As illustrated in the Supplementary Figure 1 below, the predictive performance, measured by the Area Under the Receiver Operating Characteristic Curve (AUC), was nearly identical between the two approaches:

- Complete-Case Model AUC: 0.93 (95% CI: 0.858 - 0.928)
- Imputed Data Model AUC: 0.906 (95% CI: 0.867 - 0.955)

**Supplementary Figure 1. Comparison of ROC values before and after data imputation in sensitivity analysis**

The close agreement in AUC values indicates that the multiple imputation method had a negligible impact on the model's predictive performance. This high level of consistency confirms that the core conclusions of our study are robust to the method used for handling missing data.

**References:**

[1]	Sterne, J. A., White, I. R., Carlin, J. B., Spratt, M., Royston, P., Kenward, M. G., Wood, A. M., & Carpenter, J. R. (2009). Multiple imputation for missing data in epidemiological and clinical research: potential and pitfalls. *Bmj*, *338*, b2393. <https://doi.org/10.1136/bmj.b2393>

[2]	Jakobsen, J. C., Gluud, C., Wetterslev, J., & Winkel, P. (2017). When and how should multiple imputation be used for handling missing data in randomised clinical trials - a practical guide with flowcharts. *BMC Med Res Methodol*, *17*(1), 162. <https://doi.org/10.1186/s12874-017-0442-1>
