# Supplementary material for: Development and validation of a CT-based predictive model for new vertebral compression fractures: the role of vertebral CT attenuation and paraspinal muscle
Source: Front Med (Lausanne). 2026 Jul 22;13:1860827. doi: 10.3389/fmed.2026.1860827 (PMC13437277; doi:10.3389/fmed.2026.1860827)
Supplement: Supplementary file 2 [file Data_Sheet_2.docx]

**Development and Validation of a CT-Based Predictive Model for New Vertebral Compression Fractures: The Role of Vertebral BMD and Paraspinal Muscle**

**Yiqi Wu^1^，Qing Song^1^，Canwei Hu^1^，Chaoyi Yin^1^，Zhiqing Chen^1^，Pujian Zhong^1^，Binshan Zhang^1*^**

**^1^**Guangzhou University of Traditional Chinese Medicine Dongguan Hospital, China

*** Correspondence:**
Binshan Zhang

13600298950@163.com

### Supplementary Material 2: Multicollinearity test

**Supplementary Table 1. Results of multicollinearity test**

| **Variable** | **VIF** | **1/VIF** |
| --- | --- | --- |
| Age | 2.287 | 0.437 |
| Gender | 1.884 | 0.531 |
| Low Energy Trauma | 1.350 | 0.741 |
| Smoke | 1.572 | 0.636 |
| Alcohol | 1.572 | 0.636 |
| Chronic Kidney Disease | 1.000 | 1.000 |
| Diabetes | 1.327 | 0.754 |
| Hypertension | 1.263 | 0.792 |
| Bone Cement Leaking | 1.000 | 1.000 |
| Single Segment | 4.062 | 0.246 |
| Bone Cement Volume | 4.928 | 0.203 |
| T11-L2 Fracture | 1.475 | 0.678 |
| Cr | 1.705 | 0.587 |
| UREA | 1.506 | 0.664 |
| ALB | 1.674 | 0.597 |
| AST/ALT | 1.827 | 0.547 |
| TC | 1.596 | 0.627 |
| TG | 1.514 | 0.660 |
| HDL | 1.412 | 0.708 |
| Paravertebral Muscle Area | 1.289 | 0.776 |
| T Value_Lumbar Vertebra | 1.500 | 0.667 |
| Bone CT Value | 1.645 | 0.608 |

**Abbreviations:**  VIF,Variance Inflation Factor.

**Supplementary Table 2. Multicollinearity test for predictor variables**

| **Variable** | **VIF** | **1/VIF** |
| --- | --- | --- |
| Age | 1.144 | 0.874 |
| ALB | 1.203 | 0.831 |
| Paravertebral Muscle Area | 1.077 | 0.929 |
| HU | 1.071 | 0.933 |
| Low Energy Trauma | 1.018 | 0.982 |
| Single Segment | 1.015 | 0.985 |
